# Dynamic secondary degeneration in the spinal cord and ventral root after a focal cerebral infarction among hypertensive rats

**DOI:** 10.1038/srep22655

**Published:** 2016-03-07

**Authors:** Ge Dang, Xinran Chen, Yicong Chen, Yuhui Zhao, Fubing Ouyang, Jinsheng Zeng

**Affiliations:** 1 Department of Neurology and Stroke Center, The First Affiliated Hospital, Sun Yat-Sen University, Guangzhou, Guangdong 510080, China

## Abstract

Cerebral infarction can cause secondary damage to nonischemic brain regions. However, whether this phenomenon will appear in central nervous system regions outside the brain remains unclear. Here we investigated pathological changes in the spinal cord and ventral root after ischemic stroke. All rats exhibited apparent neurological deficits post-MCAO, which improved gradually but could still be detected 12-weeks. Neuronal filaments in the corticospinal tract (CST) and neurons in the ventral horn were significantly declined in the contralateral cervical and lumbar enlargement 1-week post-MCAO. These decreases remained stable until 12-weeks, accompanied by progressively increased glial activation in the ventral horn. Axonal degeneration and structural derangement were evident in the contralateral cervical and lumbar ventral root 1-week post-MCAO; these changes spontaneously attenuated over time, but abnormalities could still be observed 12-weeks. The number of neural fibers in the contralateral CST and neurons in the contralateral ventral horn were positively correlated with neurological scores 12-weeks post-MCAO. Additionally, GFAP^+^cell density in the contralateral CST and ventral horn was negatively correlated with neurological scores. Our results suggest that cerebral infarction can elicit secondary degeneration in the cervical and lumbar spinal cord, as well as the projecting ventral root, which may hamper functional recovery after stroke.

Focal cerebral infarctions can induce trans-synaptic degeneration in nonischemic, remote brain areas, such as the thalamus, hippocampus, and substantia nigra, which have synaptic connections with primary ischemic sites^1^,^2^,^3^. Such secondary degeneration has been demonstrated with neuroimaging techniques and pathological examinations across both clinical studies and animal experiments^4^. However, more distant regions outside of the brain, including the spinal cord and ventral root, attract little attention. Whether trans-synaptic degradation is also elicited in these areas after an ischemic stroke lacks sufficient evidence^4^,^5^.

Wu and Ling^6^,^7^ reported that glial reactions and neuronal degeneration could exist in a rat’s lumbar spinal cord grey matter following a middle cerebral artery occlusion (MCAO). They also observed that lumbar ventral horn neurons remain ultra-structurally intact. A pioneering postmortem study demonstrated that upper motor neuron lesions among stroke patients did not induce neuronal degeneration in the spinal ventral horn cells^8^. Thus, secondary degeneration in the spinal cord following a cerebral infarction has yet to be confirmed. As for the peripheral nerves, previous electrophysiological studies suggested that stroke patients showed a significant decline in motor conduction velocity, compound motor action potential, and motor unit numbers in the hemiplegic limbs, suggesting axonal degeneration of the peripheral nerves^9^,^10^,^11^. Nevertheless, the lack of pathological evidence in peripheral nerves remains. Although previous studies revealed that large and myelinated fibers of the ventral root decrease, and some denervated muscle fibers remain in the involved limbs among patients with cerebrovascular disease^12^,^13^, sufficient dynamic pathological evidence of delayed peripheral nerve degeneration is absent. More importantly, no previous study has elucidated any association between secondary pathological change in the spinal cord and ventral root and functional neurological recovery after an ischemic stroke.

In order to systematically demonstrate secondary damage in the spinal cord and ventral root, and relevant functional neurological recovery after a stroke, the present study investigated dynamic pathological changes in the corticospinal tract (CST) and the ventral horn of the cervical and lumbar spinal enlargement and corresponding ventral root. We further analyzed the association between these pathological changes and functional motor deficits after a focal cerebral infarction in hypertensive rats.

## Results

### Motor impairment and cortical infarction

**Figure 1:**
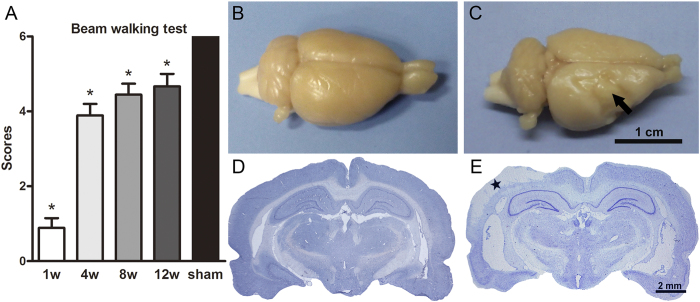
Motor function impairment and cortical infarction formation post-MCAO in hypertensive rats. (**A**) Beam walking test scores in the sham group and at W1, W4, W8, and W12 post-MCAO (n = 9, per group). (**B–E**) Gross brain morphology and Nissl staining in the sham and MCAO groups at W1 after operation. The arrow in panel C and the star in panel E indicate cortical infarction. Data are presented as medians and interquartile ranges. *p < 0.05, compared with the sham group.

All rats manifested neurological deficits at W1 post-operation, except for the sham control animals. These rats’ hemiplegic symptoms gradually improved and remained stable up to 8–12 weeks during the observation period (Fig. 1A). Gross brain morphology and Nissl staining confirmed the existence of focal cerebral infarctions in the MCAO group, which were predominantly located in the ipsilateral primary and secondary somatosensory cortices, whereas no infarctions were observed in the sham group (Fig. 1B–E).

### Neuronal degeneration and glial activation in the spinal cord

**Figure 2:**
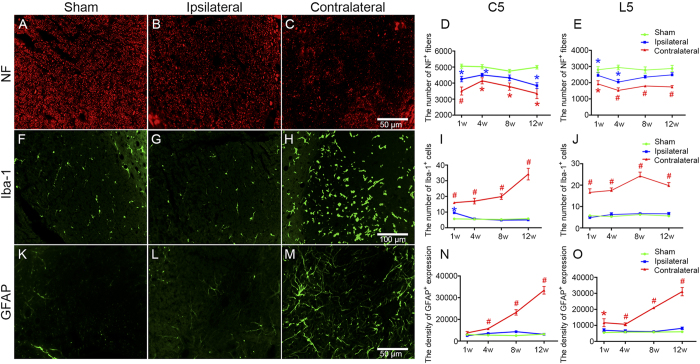
Neural filament degeneration and glial activation in the CST zone of the cervical and lumbar enlargement post-MCAO in hypertensive rats. Representative microphotographs of NF (**A–C**), Iba-1 (**F–H**), and GFAP (**K–M**) expressions in the CST zone of the cervical 5 segments at W12 post-operation in the sham, ipsilateral, and contralateral groups. Analysis of NF^+^fiber number (**D**,**E**), Iba-1^+^cell number (**I**,**J**), and GFAP^+^expression density (**N**,**O**) in the CST zone of the cervical 5 and lumbar 5 segments in the sham, ipsilateral, and contralateral groups at W1, W4, W8, and W12 post-operation (n = 6, per group at each time point). Data are presented as means ± SEM. *p < 0.05, compared with the sham group; #*p* < 0.05, compared with the sham and ipsilateral groups.

In both the cervical and lumbar enlargement, the number of NF^+^neural fibers was significantly decreased in the contralateral CST zone at W1, W4, W8, and W12 post-MCAO compared with the sham group (all *P*s < 0.05), while no significant differences were found between these time points (Fig. 2A–E). Additionally, there was an evident decline in terms of NF^+^neural filament expression in the ipsilateral CST within the cervical and lumbar enlargement at W1 and W4 post-stroke as compared to the sham controls (all *P*s < 0.05) (Fig. 2D,E). The number of Iba-1^+^microglia in the contralateral cervical and lumbar CST region increased at W1, W4, W8, and W12 post-MCAO compared with the sham group (all *P*s < 0.05), and a temporary proliferation at W1 was also observed in the ipsilateral counterpart (*P* < 0.05) (Fig. 2F–J). Astrocytes processed with GFAP labeling in the contralateral cervical and lumbar CST zone built up progressively from W1 to W12 post-MCAO, and the corresponding analyses suggested that GFAP^+^cell expression at each time point was greater than in the sham control group (all *P*s < 0.05) (Fig. 2K–O).

**Figure 3:**
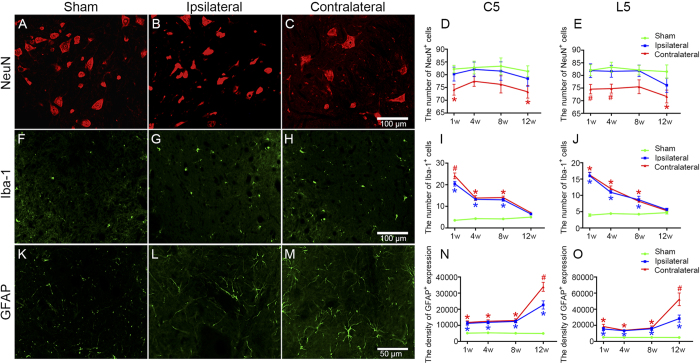
Neuronal loss and glial activation in the ventral horn of the cervical and lumbar enlargement post-MCAO in hypertensive rats. Representative microphotographs of NeuN (**A–C**), Iba-1 (**F–H**), and GFAP (**K–M**) expressions in the ventral horn of the cervical 5 segments at W12 in the sham, ipsilateral, and contralateral groups. Analysis of NeuN^+^cell number (**D**,**E**), Iba-1^+^cell number (**I**,**J**), and GFAP^+^expression density (**N**,**O**) in the ventral horn of the cervical 5 and lumbar 5 segments in the sham, ipsilateral, and contralateral groups at W1, W4, W8, and W12 (n = 6, per group at each time point). Data are presented as means ± SEM. *p < 0.05, compared with the sham group; #*p* < 0.05, compared with the sham and ipsilateral groups.

In the contralateral ventral horn of the cervical and lumbar enlargement, the number of NeuN^+^neurons declined at W1 post-MCAO compared with the ipsilateral counterpart and the sham control group (*P* < 0.05). This decrease was non-significant between these groups at W4 and W8, except for the contralateral lumbar ventral horn at W4, whereas NeuN-positive cells were reduced again in the contralateral group at W12 post-MCAO compared with the sham group (*P* < 0.05) (Fig. 3A–E). In contrast, compared to the sham group, the number of Iba-1^+^microglia increased sharply in the bilateral ventral horn at W1 post-MCAO (*P* < 0.05). The number of microglia decreased gradually at W4, W8, and W12 compared to W1; and the bilateral Iba-1-positive cells, even at W8 post-stroke, were still greater in the ventral horn of the cervical and lumbar enlargement than was the case in the sham group (*P* < 0.05). However, no significant difference was found at W12 after MCAO compared with the sham group (Fig. 3F–J). Furthermore, GFAP^+^astrocytic density increased significantly in the bilateral ventral horns of the cervical and lumbar enlargement at W1, W4, W8, and W12 post-MCAO compared with the sham group (all *P*s < 0.05). Among these time points, GFAP^+^cell expression was highest at W12 and was greater in the contralateral ventral horn than in the ipsilateral horn and the sham group (all *P*s < 0.05) (Fig. 3K–O).

### Axonal degeneration and structural derangement in the spinal ventral root

**Figure 4:**
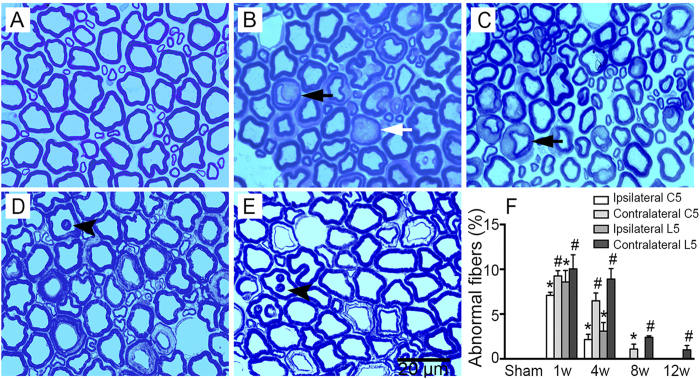
Axonal degeneration and structural disorder in the ventral root of the cervical and lumbar 5 segments post-MCAO in hypertensive rats. Representative toludine blue staining microphotographs of the contralateral ventral root of the cervical 5 segments in the sham group (**A**) and at W1 (**B**), W4 (**C**), W8 (**D**), and W12 (**E**) post-MCAO. Fewer circular axons with abnormal myelin sheaths (black arrow) were observed at W1 and W4. Darkly stained axons indicative of degeneration (white arrow) appeared at W1. Infolding of the myelin sheaths (arrowhead) was seen at W8 and W12. (**F**) Analysis of the percentage of abnormal fibers in the ventral root of the cervical 5 and lumbar 5 segments at W1, W4, W8, and W12 post-operation in the sham, ipsilateral, and contralateral groups (n = 3, per group at each time point). Data are presented as means ± SEM. *p < 0.05, compared with the sham group; #*p* < 0.05, compared with the sham and ipsilateral groups.

Toludine blue staining revealed that the cervical 5 ventral root structure was normal after the sham operation, whereas several abnormal neuronal fibers (with fewer circular axons and disordered myelin sheaths) appeared at W1 and W4 post-MCAO (Fig. 4A–C). Besides, deranged nerve fibers, characterized by infolding of the myelin sheath, could also be observed at W8 and W12 post-MCAO (Fig. 4D,E). Results suggested that, compared with the sham group, the abnormal neuronal fibers significantly increased at W1 and W4 post-stroke onset in the bilateral cervical 5 ventral root, especially on the contralateral side (Fig. 4F; all *P*s < 0.05). At W8 and W12 post-MCAO, while no abnormal fibers were found in the ipsilateral cervical 5 ventral root and the percentage of abnormal fibers had decreased on the contralateral side, this percentage was still higher than was observed in the sham group (Fig. 4F; *P* < 0.05). Similar changes were observed in the lumbar 5 ventral root (data not shown).

**Figure 5:**
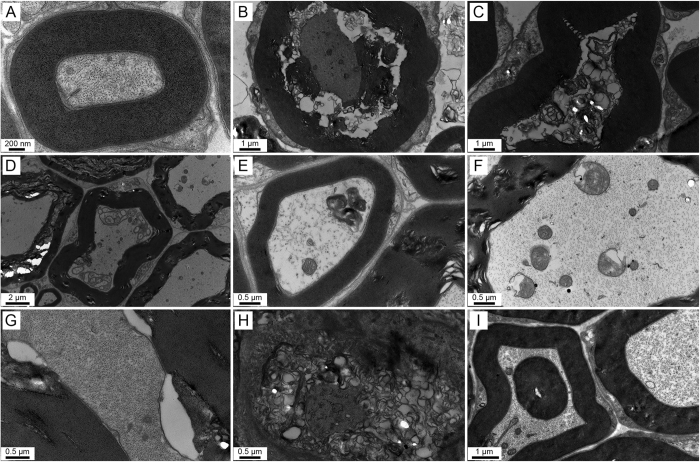
Various types of axonal degeneration and myelin sheath abnormalities detected by electron microscopy in the ventral root of the cervical 5 segment post-MCAO in hypertensive rats. (**A**) A normal axon with an intact myelin sheath. (**B**) A shrunken axon surrounded by debris from the myelin sheath. (**C**) A degenerated axon without a microfilament and microtubules. (**D**,**E**) Axoplasmic accumulation of dystrophic membranous debris with different profiles. (**F**) Swollen mitochondria in the axon. (**G**) Peri-axonal swelling. (**H**) A degenerated axon with loose myelin sheath lamellae. (**I**) Infolding of the myelin sheath. Panel A was examined at 1 week after sham operation. Panels (**B**–**H**) were observed at W1, and panel I was detected at W12 post-MCAO.

In agreement with the toludine blue staining results, various types of axonal degeneration and apparent disordered myelin sheaths in the cervical 5 ventral root were observed under the transmission electron microscope after MCAO. This was in comparison with the sham group (Fig. 5A), whereby the MCAO rats had shrunken axons, vacuole-like denaturation, demyelination, axoplasmic accumulation of dystrophic membranous debris, swollen mitochondria in the axons, peri-axonal swelling, widened spacing of the Schmidt-Lanterman incisures, axons with stripped myelin sheaths at W1 (Fig. 5B–H), or infolding of the myelin sheaths at W12 post-MCAO (Fig. 5I). Similar changes were also found in the lumbar 5 ventral root (data not shown).

### Correlations between secondary degeneration and neurological scores

Cerebral infarctions mainly lead to neurological deficits in the contralateral limb; thus, secondary degeneration on the contralateral side was chosen for our correlation analyses. Results revealed that the number of NF^+^neural fibers in the contralateral CST zone of cervical and lumbar 5, and the number of NeuN^+^neurons in the contralateral ventral horn of cervical and lumbar 5, were both positively correlated with Beam walking test scores at W12 post-MCAO (all *P*s < 0.05). Moreover, GFAP^+^cell density in the contralateral CST zone and ventral horn of lumbar 5 was negatively correlated with Beam walking test scores (*P* < 0.05). However, GFAP^+^cell density in the contralateral CST zone and ventral horn of cervical 5, the Iba-1^+^cell number in the contralateral CST zone and ventral horn of cervical and lumbar 5, and the percentage of abnormal fibers in the ventral root of cervical and lumbar 5, were not significantly correlated with Beam walking test scores at W12 post-MCAO (Table 1).

## Discussion

In the present study, dynamic pathological changes in the cervical and lumbar CST zone, ventral horn, and ventral root were systematically investigated after focal cerebral infarctions in hypertensive rats. We found that degeneration was evident in these areas post-MCAO, including neuronal filament loss, total neuronal loss, glial activation in the CST region and spinal ventral horn, and axonal degeneration and structural derangement in the ventral root. Our results suggest that cerebral infarctions can induce secondary degeneration in nervous system regions outside the brain.

Pioneering studies performed by Wu and Ling^6^,^7^ suggested that inflammatory responses, including reactions from astrocytes and microglia, were induced in a rat’s contralateral lumbar dorsal and ventral horn within 7 days post-MCAO. We also observed astrocytic activation and microglial proliferation in the bilateral cervical and lumbar ventral horn post-MCAO in our hypertensive rats. Additionally, dynamic glial reaction changes were observed, which were characterized by the number of microglia sharply increasing at W1 and gradually subsided by W12; however, astrocytic expression increased from W1 to W12 post-MCAO. These alterations are similar to results from longitudinal secondary changes in the substantia nigra post-stroke^14^. We also discovered significant neuronal loss in the contralateral cervical and lumbar ventral horn at W1 and W12 post-MCAO. However, Wu and Ling^7^ did not observe neuronal damage in the lumbar ventral horn following a stroke. They speculated that the deafferentation process, due to anterograde degeneration of the CST, was not severe enough to induce neuronal degeneration in the ventral horn. In contrast, we found a substantial decrease in neural fibers, as well as astrocytic and microglia activation, in the contralateral CST zone of the cervical and lumbar enlargement. This is consistent with a previous study showing microglial reactions in the CST at the cervical level^15^. Based on previous research, we postulated that neuronal degeneration would be observed in our study. Moreover, a previous postmortem study did not detect trans-synaptic degeneration among lumbar ventral horn neurons in a group of stroke patients^8^. One possible explanation for this discrepancy could be that while CST axon terminals end mainly in the dorsal horn and exert control over movement primarily via interneurons in humans, direct synaptic contact between CST neurons and ventral horn neurons exist in rats^16^,^17^,^18^. It has been further reported that CST neurons affected by an ischemic insult release glutamate at their terminals in the spinal cord, leading to anterograde degeneration^19^, which might further induce transneuronal damage in corresponding projected areas in both humans and rats, respectively.

Typically, electromyography is applied to evaluate post-stroke secondary changes in the peripheral nervous system^20^. Previous studies demonstrated that electrophysiological indicators, including motor conduction velocity, compound motor action potential, or motor unit number, were significantly decreased to different degrees in the hemiplegic limbs of stroke patients, suggesting axonal degeneration or demyelination of peripheral nerves^9^,^10^,^11^. However, studies assessing pathological post-stroke changes in peripheral nerves were rarely reported. In the present study, we detected apparent axonal degeneration and derangement of myelin sheaths in the contralateral cervical and lumbar ventral root post-MCAO in rats. This is in partial agreement with a pioneering morphometric study that found significant myelinated fiber loss and large fiber decreases in the lumbar ventral root among patients with cerebrovascular diseases^13^. Additionally, the pathological changes characterized by degeneration in the axon and myelin sheaths observed in our study may account for these electrophysiological alterations, including declined motor conduction velocity and compound motor action potential observed elsewhere^21^,^22^.

Astrocytes and microglia respond to diverse forms of central nervous system (CNS) injury with their morphological appearance from a so-called surveying state, characterized by a small cell body and thin processes, to an activated state, characterized by enlargement of the cell body and thickening of processes, which are collectively referred to as glial activation^23^,^24^. Glial, including astrocytic and microglial, activation served as an indicator of damage to the CNS and have been reviewed extensively elsewhere^25^,^26^,^27^. Through the production of various proinflammatory cytokines, chemokines, and different inflammatory mediators, glial activation exerts deleterious effects that can be involved in hindering axonal regeneration, interfering with synaptic plasticity, impairing white matter integrity, or impeding neurological function recovery^26^,^28^,^29^,^30^,^31^. To be honest, it is difficult to demarcate the difference in effect between astrocytic and microglial activations after ischemic stroke, which may play both detrimental and beneficial roles in the repair process and could influence each other^32^,^33^. Traditionally, activated astrocytes can be classified as anisomorphic, where astrocytes undergo morphological change, which is often associated with tissue damage, or isomorphic, whereby astrocytes preserve normal morphology and promote neurite outgrowth and facilitate synaptogenesis^34^,^35^. In addition, astrocytic activation in the acute stage after CNS injury has been reported to be beneficial and critical for recovery, whereas the presence of these activated cells in the chronic phase is inhibitory and contributes to sustained inflammation^36^,^37^. In this study, we found that the expression of activated astrocytes was significantly increased in both CST and ventral horn at 12 W after MCAO. These astrocytes inverted to an obvious ramified shape with long processes compared with the sham group (Figs 2 and 3K–O), which might account for the negative correlation between astrocytic activation and motor functional at 12 W post-MCAO (Table 1). On the other hand, the activated microglia after ischemic stroke is known to include the M1 and M2 phenotypes simultaneously, which can impede CNS repair, expand tissue damage (M1), and promote brain recovery by clearing cell debris, resolving local inflammation, and releasing trophic factors (M2), respectively^28^. The cytotoxic effects of microglial activation occur soon after stroke and can continue to exacerbate injury for a few days afterward. The later effects of activated microglia may be important for tissue repair and wound healing^38^,^39^. Moreover, in the present study, no significant difference was found in the expression of activated microglia in the ventral horn at W12 after MCAO, in comparison with the sham group (Fig. 3F–J). These reasons may explain the result that a decrease in microglial activation was not related to motor function recovery at 12 W post-MCAO (Table 1), to some extent. However, attenuation of astrocytic activation has also been reported to reduce CST axonal remodeling in the denervated spinal cord and delay neurological recovery after cerebral infarction^40^. Therefore, further studies are needed to fully understand the sophisticated dualistic role of astrocytic activation in ischemic stroke.

Although secondary degeneration was elicited in the CST zone, as well as the ventral horn of the cervical and lumbar enlargement and their corresponding ventral roots post-cerebral infarction in rats, our results demonstrated that motor functioning deficits spontaneously improved to a great extent. We suggest that the gradual attenuation of microglial activation in the contralateral cervical and lumbar ventral horn, and decreases in abnormal fibers within the contralateral cervical and lumbar ventral root, might be a potential reason for the observed motor functioning recovery observed in our study^28^. Conversely, our previous studies suggested that motor-related cortical volume increases and white matter remodeling related to motor processing could occur in patients with a cerebral infarction, and neurogenesis and angiogenesis was induced within remote regions among a sample of rats post-MCAO^41^,^42^,^43^. This might underlie the motor functioning improvement we observed, as well. Nevertheless, the current rat sample still exhibited certain neurological deficits at W12 post-MCAO compared with a sham group. Consistent with this result, secondary damage characterized by a significant loss in neuronal filaments and whole neurons, combined with glial activation in the CST region and ventral horn of the cervical and lumbar enlargement, was present. Furthermore, we revealed that the number of NF^+^neural fibers in the contralateral CST zone and NeuN^+^neurons in the contralateral ventral horn of cervical and lumbar 5 were both positively correlated with neurological scores at W12. Conversely, GFAP^+^cell density in the contralateral CST zone and ventral horn of lumbar 5 was negatively correlated with neurological scores at W12. Thus, secondary damage may impede functional recovery. Emerging studies have demonstrated that remote lesions may hamper neurological recovery and predict deficient motor outcomes after a stroke^44^,^45^,^46^. In the light of the present results, secondary degeneration in the spinal cord and ventral root caused by a cerebral infarction requires more attention for facilitating better stroke management.

In conclusion, our results demonstrate that secondary degeneration can be induced within remote nervous system regions outside of the brain after a cerebral infarction, which may hamper functional recovery after ischemic stroke. Meanwhile, a few study limitations should be noted. First, secondary damage to peripheral nerves and skeletal muscles were not investigated. Second, no underlying mechanism(s) for the revealed phenomena was explored in the present study. Further studies applying multiple neuroprotectants are warranted to elucidate the role of trans-synaptic degeneration in functional neurological recovery after a cerebral ischemia.

## Methods

### Animal model

The Institutional Animal Ethical Committee of Sun Yat-Sen University authorized the experimental protocol, and all procedures involving animals were performed in accordance with National Institute of Health guidelines for the care and use of laboratory animals. A total of 88 male Sprague-Dawley rats weighing 70–90 g were used to develop the stroke-prone renovascular hypertensive model according to a method described previously^47^. Approximately 12 weeks later, 80 rats with a systolic blood pressure higher than 180 mmHg and without spontaneous stroke symptoms were randomly assigned to receive MCAO surgery (*n* = 36) or sham operations (*n* = 36) as described previously^48^. In brief, under anesthesia with 10% chloral hydrate (3 ml/kg, intraperitoneally), the right middle cerebral artery was exposed and then occluded above the olfactory tract by bipolar electrocoagulation to establish the MCAO model. Animals that underwent all procedures described above, except for an MCAO, served as the sham group. To minimize invasiveness and operation time, tracheal intubation was not performed, and an arterial was not created. Body temperature was maintained at 37 °C on a heating pad during the surgery and recovery period. After recovery, the rats were returned to cages with *ad libitum* access to food and water. The rats in the MCAO and sham-operated group were randomly divided into four time points: week 1 (W1), week 4 (W4), week 8 (W8), and week 12 (W12) post-operation (*n* = 9 in each group). The remaining 8 rats were excluded due to subarachnoid hemorrhage or death during surgery.

### Neurological assessment

The Beam walking test was assessed by 2 observers, blinded to the experimental procedures, at W1, W4, W8, and W12 post-MCAO. Beam walking performance was assessed on a 7-point scale^49^: 0 = falling from the beam, 1 = inability to traverse the beam but maintaining balance on the beam, 2 = traversing the beam while dragging the affected limb, 3 = traversing the beam without using the affected limb, 4 = traversing the beam using the affected limb in less than half of its steps, 5 = traversing the beam using the affected limb in more than half of its steps, and 6 = traversing the beam normally.

### Tissue preparation

At W1, W4, W8, and W12 post-real or sham MCAO, 6 rats per group were deeply anesthetized with 10% chloral hydrate (5 ml/kg, intraperitoneally); the rats were transcardially perfused with 0.9% saline at 4 °C followed by 4% paraformaldehyde in 0.1 M phosphate buffered-saline (PBS, pH = 7.4). The brains were first exposed to confirm the existence of a focal cerebral infarction, and then, the cervical and lumbar enlargement of the spinal cord were removed and immersed in the same fixative followed by dehydration in 20% and 30% sucrose, sequentially. Coronal slices were cut on a cryostat (Leica, CM1900, Germany) for immunofluorescence.

The remaining 3 rats per group were perfused with 4% paraformaldehyde and 2.5% glutaraldehyde mixture fixation. The ventral root of cervical 5 and lumbar 5 were collected and post-fixed in 1% osmium tetroxide. After progressive dehydration in ethanol, samples were embedded in epoxy resin and sectioned with an ultramicrotome (Leica, UC6, Germany) for toludine blue staining and electron microscopic analysis.

### Nissl staining, Toludine blue staining and electron microscopic analysis

A set of sections was chosen for Nissl staining to evaluate the existence of infarcts. Nissl staining was carried out with 0.1% cresyl violet according to a standard procedure. Semi-thin (750 nm) and ultra-thin (80 nm) cross sections were used for toludine blue staining and electron microscopic analyses to characterize the morphological changes of the cervical and lumbar ventral root. Semi-thin sections were stained with 0.1% toluidine blue, dehydrated with ethanol, and examined under a light microscope (Olympus, BX51). Ultra-thin slices were stained in saturated uranyl acetate and lead citrate; stained sections were then observed and photographed under a transmission electron microscope (FEI, Tecnai G2 Spirit Twin).

### Immunofluorescence

A series of sections were selected for immunofluorescence to evaluate neuronal loss and gliosis in the CST zone and ventral horn. Briefly, sections were treated for 5 min with hot citrate buffer (85 °C, 0.01 m/L, pH 6.0) for post-antigen retrieval and then permeabilized with 0.3% Triton for 30 min followed by blocking with 5% normal goat serum for 1 h at room temperature. Subsequently, sections were incubated with the following primary antibodies at 4 °C overnight: mouse anti-neurofilament (NF, a marker of axons, 1:800, Sigma), mouse anti-neuronal nuclei (NeuN, a marker of neurons, 1:500, Millipore), rabbit anti-ionized calcium-binding adapter molecule 1 (Iba-1, a marker of microglia, 1:500, Wako), or rabbit anti-glial fibrillary acidic protein (GFAP, a marker of astrocytes, 1:500, Millipore). Negative control sections were incubated with 0.01 M PBS (pH = 7.4) instead of a primary antibody. After being rinsed with 0.01 M PBS for 10 min 3 times, sections were incubated for 1 h at room temperature with secondary antibodies: Alexa Fluor 488-conjugated goat anti-mouse IgG or Alexa Fluor 555-conjugated goat anti-rabbit IgG (1:1000, Cell Signaling Technology). Fluorescence signals were detected with a fluorescence microscope (BX51, Olympus).

### Image analysis and quantification

Ten consecutive sections at every 10^th^ section from each sample were analyzed by an experimenter who was blind to the experimental protocol. For quantitative analysis of the spinal cord immunostaining, immunopositive cells were counted using ImageJ software (National Institutes of Health, Bethesda, USA) in 3 non-overlapping fields (425 μm × 320 μm, 400× magnification) of each section^50^. For quantification of the ventral root, the abnormal myelin sheaths were counted using ImageJ software in 10 non-overlapping fields (60.08 ± 3.339 nerve fibers per field, 1,000× magnification) on each section.

### Statistical analyses

Statistical analyses were performed using SPSS 13.0 for windows (SPSS Inc., Chicago, IL, USA). Data were expressed as means ± standard error of mean (SEM). Statistical analyses were conducted using Students’ *t* tests for two-group comparisons or one-way analyses of variance (ANOVA) followed by Bonferroni corrections for multiple comparisons. Neurological deficit scores were compared using nonparametric Friedman tests. Spearman correlation analyses were used to determine the association between secondary degeneration and neurological deficit scores. *P* < 0.05 was considered statistically significant.

## Additional Information

**How to cite this article**: Dang, G. *et al.* Dynamic secondary degeneration in the spinal cord and ventral root after a focal cerebral infarction among hypertensive rats. *Sci. Rep.* **6**, 22655; doi: 10.1038/srep22655 (2016).

## Figures and Tables

**Table 1:** Spearman correlations between secondary degeneration and Beam walking test scores at W12 post-MCAO.

Secondary degeneration at the contralateral side at W12 post-MCAO	Beam walking test scores at W12 post-MCAO r (p)
NF in CST zone of cervical 5	0.85 (0.03)
NF in CST zone of lumbar 5	0.97 (0.00)
NeuN in ventral horn of cervical 5	0.88 (0.02)
NeuN in ventral horn of lumbar 5	0.91 (0.01)
GFAP in CST zone of cervical 5	−0.79 (0.06)
GFAP in CST zone of lumbar 5	−0.85 (0.03)
GFAP in ventral horn of cervical 5	−0.77 (0.08)
GFAP in ventral horn of lumbar 5	−0.88 (0.02)
Iba-1 in CST zone of cervical 5	−0.68 (0.14)
Iba-1 in CST zone of lumbar 5	−0.62 (0.19)
Iba-1 in ventral horn of cervical 5	−0.50 (0.31)
Iba-1 in ventral horn of lumbar 5	−0.53 (0.28)
Abnormal fiber in ventral root of cervical 5	−0.87 (0.33)
Abnormal fiber in ventral root of lumbar 5	−0.87 (0.33)
